# Air Pollution Exposure and Physical Activity in China: Current Knowledge, Public Health Implications, and Future Research Needs

**DOI:** 10.3390/ijerph121114887

**Published:** 2015-11-20

**Authors:** Jiaojiao Lü, Leichao Liang, Yi Feng, Rena Li, Yu Liu

**Affiliations:** 1 Key Laboratory of Exercise and Health Sciences of Ministry of Education, Shanghai University of Sport, Shanghai 200438, China; E-Mails: ljj27@163.com (J.L.), liangleichao2010@163.com (L.L.), soleilfeng@sina.com (Y.F.); 2 Center for Hormone Advanced Science and Education, Roskamp Institute, Sarasota, FL 34243, USA

**Keywords:** physical activity, health promotion, air pollution, public health, China

## Abstract

Deteriorating air quality in China has created global public health concerns in regard to health and health-related behaviors. Although emerging environmental regulations address ambient air pollution in China, the level of enforcement and long-term impact of these measures remain unknown. Exposure to air pollution has been shown to lead to multiple adverse health outcomes, including increased rates of heart disease and mortality. However, a lesser-known but increasingly significant concern is the relationship between air pollution and its effects on outdoor exercise. This is especially important in China, which has a culturally rooted lifestyle that encourages participation in outdoor physical activity. This article evaluates the intersection of air pollution and outdoor exercise and provides a discussion of issues related to its public health impact in China, where efforts to promote a healthy lifestyle may be adversely affected by the ambient air pollution that has accompanied rapid economic development and urbanization.

## 1. Introduction

In recent years, China’s ever-increasing air pollution problem has attracted worldwide attention. Air pollution commonly refers to the concentration of harmful emissions in the environment, usually from heavy traffic, industry, power generation, and coal smoke. These pollutants, induced by human activity, can have a major impact on public health, and international government agencies and health organizations, including the World Health Organization (WHO) and the Chinese government, have provided specific air quality guidelines to limit human exposure to ambient air pollutants such as particulate matter (soot), ozone, and nitrogen dioxide (see Table 1) [1,2,3,4]. However, meeting these guidelines for improving urban air quality presents a great challenge in China, which remains in the midst of a rapid expansion of economic development and urbanization. 

**Table 1:** Current guidelines for air pollutant exposure limits.

Pollutant	WHO [1]		US [2]		EU [3]		China [4]	
	Annual	24 h	Annual	24 h	Annual	24 h	Annual	24 h
PM_2.5_	10	25	12	35	12	25	15	35
PM_10_	20	50			20	25	40	50
SO_2_		20			8	50	40	80
NO_2_	40						20	50

Notes: PM_2.5_ = Particulate matter with a diameter of 2.5 micrometers, measured in µg/m^3^; PM_10_ = Particulate matter with a diameter of 10 micrometers, measured in µg/m^3^; SO_2_ = Sulfur dioxide, measured in µg/m^3^; NO_2_ = Nitrogen dioxide, measured in µg/m^3^.

**Figure 1:**
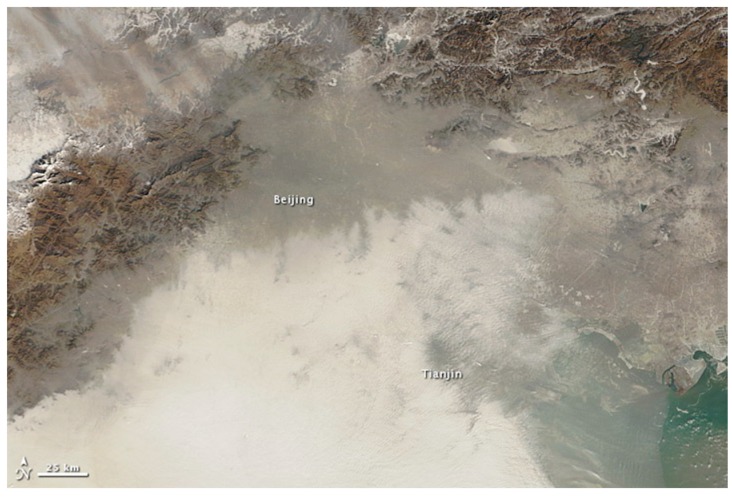
A satellite image taken on 14 January 2013. There was extensive haze, low clouds, and fog over the Beijing and Tianjin region. The brightest areas tend to be clouds or fog, which have a tinge of gray or yellow from the air pollution. Ground-based sensors at the U.S. Embassy in Beijing reported a PM_2.5_ concentration of 291 μg/m^3^ of air on this day. Source: http://earthobservatory.nasa.gov/IOTD/view.php?id=80152.

As an indication of the seriousness of air pollution in major cities in China, including Beijing, Shanghai, Guangzhou, and Xi’an, PM_2.5_ air pollution has routinely exceeded the current guidelines. For example, air pollutant concentrations in most major urban areas in 2013 exceeded WHO guidelines [5] by four times or more the average annual limit for PM_2.5_ and by two times or more for PM_10_ (see Table 2). In the same year, 92% of 74 cities in China failed to meet China’s national ambient air quality standards [6]. In some cities, PM_2.5_ values have reached extremely hazardous levels. For example, on 12 January 2013, data on daily PM_2.5_ concentration levels measured by the U.S. Embassy’s Beijing Air Quality Monitor registered an unprecedented 886 μg/m^3^ [7], a value 35 times higher than the guideline limit set by WHO [1]. A satellite image taken by NASA two days after the peak recording is shown in Figure 1. Such hazardous levels, as determined by the Air Quality Index set by the U.S. Environmental Protection Agency (EPA) [2], are likely to affect the entire Chinese population. In terms of the impact on public health, the worsening pollution situation in China has been described as “more horrible” than the SARs epidemic in 2002 [8], given that it is likely to increase cardiovascular and cerebrovascular health risks among China’s total population [9,10]. According to estimates released from the Global Burden of Disease Study [11], ambient air pollution contributed to more than 1.3 million premature deaths in China in 2010, ranking it first among 15 countries in terms of premature mortality attributable to air pollution, with the preventable death rate being higher in megacities such as Beijing, Tianjin, Chengdu, and Shanghai [12]. Ultimately, cities such as Beijing may become “uninhabitable for human beings” [13].

**Table 2:** Annual average concentrations of air pollutants in selected major Chinese cities in 2013.

City	PM_2.5_	PM_10_	SO_2_	NO_2_
Beijing	89	108	26	56
Tianjin	96	150	59	54
Harbin	81	119	44	56
Shanghai	62	84	24	48
Nanjing	78	137	37	55
Hefei	88	119	44	56
Wuhan	94	124	33	60
Changsha	83	94	33	46
Guangzhou	53	72	20	52
Chongqing	70	106	32	38
Chengdu	96	150	31	63
Xi’an	105	189	46	57

Notes: PM_2.5_ = Particulate matter with a diameter of 2.5 micrometers, measured in µg/m^3^; PM_10_ = Particulate matter with a diameter of 10 micrometers, measured in µg/m^3^; SO_2_ = Sulfur dioxide, measured in µg/m^3^; NO_2_ = Nitrogen dioxide, measured in µg/m^3^.

Ambient air pollution has long been recognized as having adverse health effects on humans. Epidemiological studies have shown that it is associated with increased risk of developing respiratory and cardiovascular diseases, increased incidence of lung cancer and pneumonia, and increased mortality [14,15,16,17]. However, neither the extraordinary magnitude and severity of China’s air pollution nor the extent to which it impacts human health has been fully examined or understood. Of particular public health concern is the adverse health impact it has on millions of people who routinely engage in outdoor physical activity for the purpose of maintaining fitness and health [18]. 

A previous paper [18] highlighted this issue and emphasized the importance of developing public health measures and surveillance systems to maximize the safety of outdoor physical activity in a polluted environment. The present article provides an update and review of the research into the association between air pollution and physical activity and its impact on health, as well as proposing future research projects to better understand these relationships. It concludes with a brief discussion of the implications of air pollution for public health and the promotion of physical activity in polluted environments.

## 2. Physical Activity and Air Pollution Exposure

### 2.1. Health Significance of Air Pollution Exposure while Exercising Outdoors 

Engaging in physical activity requires oxygen intake, with the demand for oxygen increasing as exercise intensity increases. Breathing through the mouth, which becomes more common with exertion, bypasses nasal filtration mechanisms and both increases the amount of pollution inhaled and the degree to which it travels into the respiratory system. Thus, exercising in a polluted environment (e.g., one with high vehicular traffic) exposes individuals, especially sensitive ones, such as asthmatics, children, and the elderly, to combustion-related airborne pollutants (e.g., nitrogen oxides, carbon monoxide, PM, and ozone). Such exposure can inflame airways and worsen asthmatic responses [19,20,21,22] and trigger health problems or exacerbate existing ones such as asthma [23], cardiovascular disease (including myocardial infraction [24]), and cancer [25], leading to premature death [26,27].

Therefore, although exercise is known to be highly beneficial to health [28,29,30,31,32,33,34], engaging in it in a polluted environment may increase population-wide health risks [25]. In some cases the risks may outweigh the benefits because of the potential for long-term deleterious effects on health. The fact that most recreational physical activities in China are done outdoors [35] has made exposure to ambient air pollution a significant public health issue [18].

### 2.2. Potential Health Risks and Benefits of Physical Activity due to Ambient Air Pollution Exposure

Increased respiratory uptake and deposition of air pollutants in the lungs due to higher minute ventilation during physical activity may amplify the adverse effects of air pollution on health [25]. Several studies [22,23,36,37,38,39] have documented evidence of acute deleterious health effects of physical activity with even short-term exposure to air pollution. For example, adults with asthma who walked on a busy street in London for only two hours were found to have reduced lung function [36]. Similarly, young children engaged in high levels of exercise (*i.e*., participating in more than three athletic activities) in communities with high concentrations of ozone were shown to have a higher incidence of asthma compared to children who exercised in low-ozone communities [23]. Reduced lung function has been observed among runners after they ran near busy highways [37] and among cyclists after they rode along heavy traffic routes during rush-hour traffic [39]. 

In contrast to findings that show a simple negative relationship between exercise and air pollution, a number of studies suggest more complex relationships, including physical activity modifying or attenuating the health risks of air pollution exposure or pollution undermining the health benefits of activity. For example, a study by Yu *et al.* [40] showed that habitual physical exercise among children 8 to 12 years old in a low-pollution district improved cardiopulmonary fitness in regard to maximal oxygen uptake, but this beneficial effect was not observed among children living in a high-pollution area.

Two recent experimental studies [41,42] conducted in Barcelona (Spain), examined interactions between short-term effects of traffic-related air pollution (2 h in exposure duration) and intermittent moderate physical activity (consisting of 15-min intervals of alternating rest and cycling on a stationary bicycle) on heart and lung function in healthy individuals. Results on arterial blood pressure responses showed that physical activity weakened air-pollution-induced increases in systolic blood pressure, with a stronger effect under a low-air-pollution exposure condition [41]. Additionally, respiratory and inflammatory responses indicated that while air pollution increased negative aspects of both, exercising mitigated these changes [42]. 

With respect to mortality risk, an observational study conducted in Hong Kong revealed a similar complex relationship in that sedentary older adults with acute exposure to air pollution had a higher mortality risk attributable to the exposure compared to those who engaged in habitual physical activity [43]. The study also showed that older adults who engaged in a moderate level of exercise had lower mortality risk compared to those who engaged in a high level of exercise. In a recent large-scale prospective cohort study, Andersen *et al.* [44] showed that long-term exposure to nitrogen dioxide (NO_2_) among urban individuals between 50 and 65 years of age in Denmark modified beneficial health effects of sports participation on mortality, with stronger effects on individuals living with moderate to low levels of exposure to air pollution (*i.e*., less than 19.0 µg/m^3^ in NO_2_ exposure). Collectively, findings from these studies suggest that exercising in a polluted environment may not be completely detrimental to health, and, in some cases, the benefits of physical activity may outweigh the risks related to air pollution exposure. 

## 3. Future Research Needs

Despite the clear evidence that exposure to air pollution is detrimental to health [26,27], findings from epidemiological studies on the relationship between air pollution and physical activity are mixed, with some evidence that the health benefits of physical activity may not be adversely affected by exposure to air pollution [40,41,42,44]. The current key issue is the unknown demarcation point between the level of pollution at which physical activity can attenuate health risks associated with pollution and the level at which physical activity increases negative health outcomes due to pollution.

Given the unprecedented situation in China in terms of the extent of its pollution problem and its high population density, the lack of population-based epidemiological studies that link ambient air pollution, physical activity/inactivity, and health is extremely problematic. In this regard, research that addresses context-specific effects of air pollution on physical activity in China is urgently needed. 

First, air pollution in China is considered to be extreme because of the country’s exceptional scale of industry. Since most studies on physical activity and pollution have been conducted in developed countries where air pollution is far less severe, the extent to which those findings are applicable to China is unclear. Therefore, it is critical that an assessment of the severity of negative health consequences or physical activity effects resulting from acute and chronic exposure to air pollutants be undertaken in China.

Next, as air pollution continues to worsen in China, research is needed to explore the relationship between changes in health outcomes (positive and/or negative), total time engaged in physical activity, and level of pollution. Although animal-based research shows long-term protective effects of exercise on mice [45], there have been no human studies that help determine the critical time point at which continued or cumulative exposure to air pollution while exercising leads to a decrement in health outcomes. Future research should focus on mechanisms underlying the relationship between exercise and optimal health outcomes as modified or mediated by either acute or chronic exposure to various levels of air pollutants.

With the implementation of environmental laws by the Chinese government [46], epidemiological studies are needed to assess whether associations between long- and short-term concentrations of air pollution and indicators of health risks can be modified by levels and types of physical activity, as well as the locations (e.g., streets, residential areas) where physical activity is performed [47]. Given that air pollutant levels may change due to seasonal weather or climate changes, studies are needed to understand how the level of exposure (low, medium, high) impacts physical activity in the general population as well in “sensitive” populations involving children, older adults, and people with existing chronic medical conditions [47]. Conversely, as shown by Wong *et al.* [43], research is needed to delineate the optimal level or threshold effect of physical activity at which it can be protective against air-pollution-related health risks or mortality. 

Acquiring knowledge in these areas is important from the perspectives of public health and physical activity promotion because air pollution discourages people from engaging in regular outdoor physical activities [48,49,50]. Evidence-based knowledge is also important to inform decision-making by government and public health authorities in developing guidelines and intervention strategies to promote active lifestyles while reducing the risks from exposure to air pollution [47].

## 4. Public Health Implications

Despite the known adverse health impact of air pollution, current evidence from epidemiological and ecological-based studies suggests that engaging in physical activity in a polluted-air environment may not diminish the positive effects of exercise and should not be completely avoided in such environments. Moderate levels of physical activity, such as habitual walking or cycling, may still be beneficial for healthy individuals if done where the concentration of airborne pollution is low [41,44]. However, evidence is lacking on the balance between the health risks and benefits at different pollution levels and in diverse populations (e.g., healthy individuals *vs.* those who are susceptible to certain diseases), indicating that different strategies may be needed for subpopulations that face greater health risks. 

The fact that engaging in physical activity in outdoor settings [35,51] is a tradition in China makes it important to consider environmental modifications and protective strategies for exercise. Certain air pollutants, such as PM, are modifiable, and interventions can therefore be developed to mitigate the level of air pollution. One strategy to offset the great expansion of urbanization is to develop green spaces that can counter the health hazards induced by air pollution [52] and then promote behaviors such as physical activity in these green spaces [53,54]. With increasing dependence on automobiles in China, encouraging community walking for utilitarian purposes becomes increasingly important for maintaining healthy lifestyles, providing health benefits, and helping reduce motorized traffic and its harmful emissions of NO_2_ [55,56,57]. 

Finally, habitual exercisers must be educated in precautions against pollution by staying away from heavy traffic or industrial areas [37] or by eliminating exercise when pollution levels are known to be high [37]. Research shows that regular and timely air quality alerts are important for informing the public about harmful air pollution and for helping people make decisions about engaging in physical activities [48]. Other protective strategies that can offset adverse health effects of air pollution include taking antioxidant supplements, wearing a facemask, and avoiding high-traffic areas [58]. A final untapped area is the provision of education and information for health care providers about potential risks for exercising outdoor on highly polluted days, especially for patients with existing medical conditions. 

## 5. Conclusions 

Air pollution in China poses a major public health threat to the promotion of physical activity. In the absence of the enforcement of meaningful environmental regulations to temper growing industrialization and urbanization, air quality will continue to deteriorate, exposing exercisers to higher levels of toxic air pollutants. Thus, large-scale public health initiatives, including expanded epidemiological research, are needed to protect the population and the long-standing Chinese tradition of exercising outdoors. 

## References

[1] World Health Organization Ambient (Outdoor) Air Quality and Health. http://www.who.int/mediacentre/factsheets/fs313/en/.

[2] U.S. Environmental Protection Agency National Ambient Air Quality Standards (NAAQS). http://www.epa.gov/air/criteria.html.

[3] EUR-Lex (2008). Directive 2008/50/EC of the European Parliament and of the Council of 21 May 2008 on Ambient Air Quality and Cleaner Air for Europe. Official J. Eur. Union.

[4] Ministry of Environmental Protection of the People’s Republic of China Ambient Aair Quality Standards. http://kjs.mep.gov.cn/hjbhbz/bzwb/dqhjbh/dqhjzlbz/201203/t20120302_224165.htm.

[5] National Bureau of Statistics and Ministry of Environment Protection of China China Environmental Sstatistical Yearbook, 2014, Table 8–19. http://www.stats.gov.cn/tjsj/ndsj/2014/indexeh.htm.

[6] Greenpeace China City Rrankings Released for PM_2.5_ Pollution in 2013. http://www.greenpeace.org/china/zh/news/releases/climate-energy/2014/01/PM25-ranking/#ednref2.

[7] U.S. Embassy in Beijing Beijing—Historical Data. http://www.stateair.net/web/historical/1/1.html.

[8] Caijing China’s Haze “More Horrible” than SARS Epidemic, Expert Warns. http://englishcaijingcomcn/2013–01–31/112478574html.

[9] Zhang L.W., Chen X., Xue X.D., Sun M., Han B., Li C.P., Ma J., Yu H., Sun Z.R., Zhao L.J. (2014). Long-term exposure to high particulate matter pollution and cardiovascular mortality: A 12-year cohort study in four cities in northern China. Environ. Int..

[10] Guo Y., Li S., Tian Z., Pan X., Zhang J., Williams G. (2013). The burden of air pollution on years of life lost in Beijing, China, 2004–08: Retrospective regression analysis of daily deaths. BMJ.

[11] Lim S.S., Vos T., Flaxman A.D., Danaei G., Shibuya K., Adair-Rohani H., Amann M., Anderson H.R., Andrews K.G., Aryee M. (2012). A comparative risk assessment of burden of disease and injury attributable to 67 risk factors and risk factor clusters in 21 regions, 1990–2010: A systematic analysis for the global burden of disease study 2010. Lancet.

[12] Lelieveld J., Evans J.S., Fnais M., Giannadaki D., Pozzer A. (2015). The contribution of outdoor air pollution sources to premature mortality on a global scale. Nature.

[13] South China Morning Pollution Makes Beijing almost ‘Uninhabitable for Human Beings’. http://www.scmp.com/news/china/article/1426587/pollution-makes-beijing-almost-uninhabitable-human-beings.

[14] Newby D.E., Mannucci P.M., Tell G.S., Baccarelli A.A., Brook R.D., Donaldson K., Forastiere F., Franchini M., Franco O.H., Graham I. (2015). Expert position paper on air pollution and cardiovascular disease. Euro. Heart J..

[15] Miller K.A., Siscovick D.S., Sheppard L., Shepherd K., Sullivan J.H., Anderson G.L., Kaufman J.D. (2007). Long-term exposure to air pollution and incidence of cardiovascular events in women. N. Engl. J. Med..

[16] Pope C.A., Ezzati M., Dockery D.W. (2009). Fine-particulate air pollution and life expectancy in the United States. N. Engl. J. Med..

[17] Pope C.A., Burnett R.T., Thun M.J., Calle E.E., Krewski D., Ito K., Thurston G.D. (2002). Lung cancer, cardiopulmonary mortality, and long-term exposure to fine particulate air pollution. JAMA.

[18] Li F., Liu Y., Lu J., Liang L., Harmer P. (2015). Ambient air pollution in china poses a multifaceted health threat to outdoor physical activity. J. Epidemiol. Community Health.

[19] Zhao Z., Chen R., Lin Z., Cai J., Yang Y., Yang D., Norback D., Kan H. (2015). Ambient carbon monoxide associated with alleviated respiratory inflammation in healthy young adults. Environ. Pollut..

[20] Rundell K.W., Spiering B.A., Baumann J.M., Evans T.M. (2005). Bronchoconstriction provoked by exercise in a high-particulate-matter environment is attenuated by montelukast. Inhal. Toxicol..

[21] Flouris A.D., Metsios G.S., Jamurtas A.Z., Koutedakis Y. (2010). Cardiorespiratory and immune response to physical activity following exposure to a typical smoking environment. Heart.

[22] Korrick S.A., Neas L.M., Dockery D.W., Gold D.R., Allen G.A., Hill L.B., Kimball K.D., Rosner B.A., Speizer F.E. (1998). Effects of ozone and other pollutants on the pulmonary function of adult hikers. Environ. Health Perspect..

[23] McConnell R., Berhane K., Gilliland F., London S.J., Islam T., Gauderman W.J., Avol E., Margolis H.G., Peters J.M. (2002). Asthma in exercising children exposed to ozone: A cohort study. Lancet.

[24] Nawrot T.S., Perez L., Künzli N., Munters E., Nemery B. (2011). Public health importance of triggers of myocardial infarction: A comparative risk assessment. Lancet.

[25] Giles L.V., Koehle M.S. (2014). The health effects of exercising in air pollution. Sports Med..

[26] Beelen R., Raaschou-Nielsen O., Stafoggia M., Andersen Z.J., Weinmayr G., Hoffmann B., Wolf K., Samoli E., Fischer P., Nieuwenhuijsen M. (2014). Effects of long-term exposure to air pollution on natural-cause mortality: An analysis of 22 European cohorts within the multicentre escape project. Lancet.

[27] Hoek G., Krishnan R.M., Beelen R., Peters A., Ostro B., Brunekreef B., Kaufman J.D. (2013). Long-term air pollution exposure and cardio-respiratory mortality: A review. Environ. Health.

[28] Centers for Disease Control and Prevention The Benefits of Physical Activity. http://www.cdc.gov/physicalactivity/everyone/health.

[29] US Department of Health and Human Services 2008 Physical Activity Guidelines for Americans. http://www.health.gov/paguidelines/pdf/paguide.pdf.

[30] World Health Organization Step to Health: A European Framework to Promote Physical Aactivity for Health. http://www.euro.who.int/__data/assets/pdf_file/0020/101684/E90191.pdf.

[31] Heath G.W., Parra D.C., Sarmiento O.L., Andersen L.B., Owen N., Goenka S., Montes F., Brownson R.C. (2012). Evidence-based intervention in physical activity: Lessons from around the world. Lancet.

[32] Khan K.M., Thompson A.M., Blair S.N., Sallis J.F., Powell K.E., Bull F.C., Bauman A.E. (2012). Sport and exercise as contributors to the health of nations. Lancet.

[33] Williams P.T. (2013). Dose-response relationship of physical activity to premature and total all-cause and cardiovascular disease mortality in walkers. PLoS ONE.

[34] Yates T., Haffner S.M., Schulte P.J., Thomas L., Huffman K.M., Bales C.W., Califf R.M., Holman R.R., McMurray J.J., Bethel M.A. (2014). Association between change in daily ambulatory activity and cardiovascular events in people with impaired glucose tolerance (navigator trial): A cohort analysis. Lancet.

[35] The Central People’s Government of the People’s Republic of China Physical Activity Survey of Urban and Rural Areas in China in 2007. http://www.gov.cn/test/2012–04/19/content_2117453.htm.

[36] McCreanor J., Cullinan P., Nieuwenhuijsen M.J., Stewart-Evans J., Malliarou E., Jarup L., Harrington R., Svartengren M., Han I.K., Ohman-Strickland P. (2007). Respiratory effects of exposure to diesel traffic in persons with asthma. N. Engl. J. Med..

[37] Rundell K.W., Slee J.B., Caviston R., Hollenbach A.M. (2008). Decreased lung function after inhalation of ultrafine and fine particulate matter during exercise is related to decreased total nitrate in exhaled breath condensate. Inhal. Toxicol..

[38] Zhang J., McCreanor J.E., Cullinan P., Chung K.F., Ohman-Strickland P., Han I.-K., Järup L., Nieuwenhuijsen M. (2009). Health effects of real-world exposure to diesel exhaust in persons with asthma. Res. Rep. Health Eff. Inst..

[39] Strak M., Boogaard H., Meliefste K., Oldenwening M., Zuurbier M., Brunekreef B., Hoek G. (2010). Respiratory health effects of ultrafine and fine particle exposure in cyclists. Occup. Environ. Med..

[40] Yu I.T., Wong T.W., Liu H.J. (2004). Impact of air pollution on cardiopulmonary fitness in schoolchildren. J. Occup. Environ. Med..

[41] Kubesch N., de Nazelle A., Guerra S., Westerdahl D., Martinez D., Bouso L., Carrasco-Turigas G., Hoffmann B., Nieuwenhuijsen M.J. (2015). Arterial blood pressure responses to short-term exposure to low and high traffic-related air pollution with and without moderate physical activity. Euro J. Prev. Cardiology.

[42] Kubesch N.J., de Nazelle A., Westerdahl D., Martinez D., Carrasco-Turigas G., Bouso L., Guerra S., Nieuwenhuijsen M.J. (2015). Respiratory and inflammatory responses to short-term exposure to traffic-related air pollution with and without moderate physical activity. Occup. Environ. Med..

[43] Wong C.M., Ou C.Q., Thach T.Q., Chau Y.K., Chan K.P., Ho S.Y., Chung R.Y., Lam T.H., Hedley A.J. (2007). Does regular exercise protect against air pollution-associated mortality?. Prev. Med..

[44] Andersen Z.J., de Nazelle A., Mendez M.A., Garcia-Aymerich J., Hertel O., Tjonneland A., Overvad K., Raaschou-Nielsen O., Nieuwenhuijsen M.J. (2015). A study of the combined effects of physical activity and air pollution on mortality in elderly urban residents: The Danish diet, cancer, and health cohort. Environ. Health Perspect..

[45] Vieira R.D.P., Toledo A.C., Silva L.B., Almeida F.M., Damaceno-Rodrigues N.R., Caldini E.G., Santos A.B.G., Rivero D.H., Hizume D.C., Lopes F. (2012). Anti-inflammatory effects of aerobic exercise in mice exposed to air pollution. Med. Sci. Sports Exerc..

[46] National People’s Congress of the People’s Republic of China China’s Legislature Adopts Rrevised Environmental Protection Law. http://www.npc.gov.cn/englishnpc/news/Legislation/2014–04/25/content_1861275.htm.

[47] Boehmer T.K. Physical Activity and Air Pollution Exposure. http://www3.epa.gov/airnow/2014conference/Plenary/Monday/Boehmer_NAQC_2014_final2.pdf.

[48] Wen X.J., Balluz L., Mokdad A. (2009). Association between media alerts of air quality index and change of outdoor activity among adult asthma in six States, BRFSS, 2005. J. Community Health.

[49] Roberts J.D., Voss J.D., Knight B. (2014). The association of ambient air pollution and physical inactivity in the United States. PLoS ONE.

[50] An R., Xiang X. (2015). Ambient fine particulate matter air pollution and leisure-time physical inactivity among US adults. Public Health.

[51] Zhang L., Zheng X. (2006). Ageing population’s sports and population health. J. Sports Sci..

[52] Greenspace Scotland Health Impact Assessment of Greenspace: A Guide. http://greenspacescotland.org.uk/health-impact-assessment.aspx.

[53] Mitchell R., Popham F. (2008). Effect of exposure to natural environment on health inequalities: An observational population study. Lancet.

[54] Bell J.F., Wilson J.S., Liu G.C. (2008). Neighborhood greenness and 2-year changes in body mass index of children and youth. Am. J. Prev. Med..

[55] Mueller N., Rojas-Rueda D., Cole-Hunter T., de Nazelle A., Dons E., Gerike R., Gotschi T., int Panis L., Kahlmeier S., Nieuwenhuijsen M. (2015). Health impact assessment of active transportation: A systematic review. Prev. Med..

[56] Frank L.D., Engelke P. (2005). Multiple impacts of the built environment on public health: Walkable places and the exposure to air pollution. Int. Reg. Sci. Rev..

[57] Marshall J.D., Brauer M., Frank L.D. (2009). Healthy neighborhoods: Walkability and air pollution. Environ. Health Perspect..

[58] Laumbach R., Meng Q., Kipen H. (2015). What can individuals do to reduce personal health risks from air pollution?. J. Thorac. Dis..

